# Entomophagy: Nutritional Value, Benefits, Regulation and Food Safety

**DOI:** 10.3390/foods14132380

**Published:** 2025-07-04

**Authors:** Noélia A. Pinheiro, Liliana J. G. Silva, Angelina Pena, André M. P. T. Pereira

**Affiliations:** LAQV, REQUIMTE, Laboratory of Bromatology and Pharmacognosy, Faculty of Pharmacy, University of Coimbra, Polo III, Azinhaga de Stª Comba, 3000-548 Coimbra, Portugal; noeliananet@gmail.com (N.A.P.); apena@ci.uc.pt (A.P.); andrepereira@ff.uc.pt (A.M.P.T.P.)

**Keywords:** regulatory framework, health benefits, food safety, food hazards, nutritional composition

## Abstract

The consumption of insects as food is an ancient practice that remains widespread in many regions of Asia, Africa, Latin America, and Oceania. However, this tradition has largely disappeared in Western countries, where it is often met with aversion. Nutritionally, insects can contain up to 60–70% protein (dry weight), along with beneficial fats, minerals, and vitamins, making them comparable to commonly consumed protein sources. Additionally, they contain bioactive compounds that offer health benefits and can contribute to reducing social inequalities in food access. As a sustainable protein source, insects have the potential to meet the demands of a projected global population of 9.7 billion by 2050. From a regulatory perspective, legislation on edible insects is still emerging in many parts of the world, with significant regional differences in the approval process, safety requirements, and permitted species. However, ensuring their safety—particularly in terms of production, preservation, storage, and potential health risks—is crucial. By addressing these concerns, it may be possible to shift the prevailing negative perception in Western societies and enhance consumer acceptance. Thus, we performed a literature review encompassing several issues regarding entomophagy, like insects’ nutritional composition, legislation, benefits, and food safety, and also addressing future perspectives.

## 1. Introduction

Entomophagy—the practice of consuming insects as food—has recently garnered increasing interest in various countries. Although often perceived as novel, it is, in fact, an ancient practice with historical roots tracing back to the 8th century BCE. Today, more than 2200 species of edible insects are recognised, serving as a regular food source for approximately two billion people worldwide [1,2,3].

Since the end of the Second World War, the global population has grown substantially. According to recent projections by the United Nations, the world population is expected to reach 9.7 billion by 2050, a scenario that presents major obstacles to achieving food security and sustainable development [4].

The current global food system is marked by inefficiencies and poses considerable threats to environmental integrity, public health, and the achievement of sustainability goals. In this context, edible insects emerge as a promising alternative or complementary source of animal protein, offering numerous advantages over conventional protein sources [5].

Disparities in global food production and consumption further exacerbate these challenges. Protein intake varies markedly across regions, with individuals in developed countries consuming, on average, 40 g more protein daily than those in developing nations. As the demand for affordable and sustainable protein continues to rise, traditional animal-based sources are becoming increasingly costly and less accessible in certain regions [5].

Currently, meat and fish remain the primary sources of protein in most countries. However, ongoing demographic growth is projected to drive up their consumption and, consequently, their production [1,6]. This surge in demand leads to the overexploitation of natural resources—such as land, water, and energy—and contributes significantly to deforestation and greenhouse gas emissions, particularly from livestock [5,7,8,9]. These environmental pressures underscore the urgent need to adopt innovative agricultural practices and shift towards healthier, more sustainable dietary patterns. Among these strategies, integrating alternative protein sources, including edible insects, can play a pivotal role in reducing the ecological footprint of food production [1].

From a nutritional standpoint, many edible insect species exhibit high protein content and provide nearly all essential amino acids, along with a rich array of micronutrients [10,11]. In addition to proteins, insect-derived carbohydrates are primarily found in the form of chitin and glycogen [12]. Chitin, a long-chain polymer of N-acetyl-D-glucosamine, forms the main component of insect exoskeletons, while glycogen serves as an energy reserve, particularly during metabolic processes such as metamorphosis [1,12,13].

Furthermore, edible insects contain bioactive compounds associated with various health-promoting properties [11]. Compared to conventional livestock, these production systems demand considerably fewer resources, utilising less land, water, and feed and generating lower greenhouse gas emissions [5]. Beyond environmental benefits, insect farming offers economic opportunities, particularly for low-income communities, and represents a growing business sector in Western markets [14].

Despite these advantages, the widespread adoption of entomophagy, especially in Western societies, faces considerable challenges [1,2]. Chief among them are concerns over food safety, encompassing production, storage, and potential contamination with biological or chemical agents, as well as allergenic and anti-nutritional factors [1,2]. Cultural taboos and consumer aversion—often stemming from perceptions of insects as unclean or primitive food—further hinder acceptance. To address these barriers, many companies are developing insect-based products in more familiar and palatable formats, aiming to increase consumer acceptance and integrate insects into mainstream diets [1,2].

Therefore, it is important to evaluate the advantages and disadvantages of this recently introduced food type in the European market impartially and determine whether it can become a viable alternative to our current food system.

This review aims to provide a comprehensive evaluation of the current scientific literature on the multifaceted practice of entomophagy, examining both its potential benefits and associated risks. Key topics addressed include the nutritional composition of edible insects, regulatory frameworks, health and environmental benefits, food safety considerations, and future prospects. By exploring these dimensions, the review seeks to offer insights into the growing global interest in entomophagy as a sustainable food source.

## 2. Methodology

The available scientific literature was searched using the PubMed, Science Direct, and Google Scholar databases, complemented by targeted searches on institutional and regulatory websites such as those of the European Food Safety Authority (EFSA), the U.S. Food and Drug Administration (FDA), Health Canada, and the Food and Agriculture Organization (FAO). The research process took place between 8 January and 27 July 2024.

To identify relevant studies, combinations of at least two of the following keywords were employed: “Entomophagy”, “Edible Insects”, “Nutritional Composition”, “Benefits”, “Food Security”, “Legislation”, “Food Safety”, “Farming”, and “Hazards”. Boolean operators (AND, OR) were used to expand or refine the search results where appropriate.

Studies were included based on their publication dates, with preference given to those from the past five years. Priority was also given to research covering multiple nationalities or regions to ensure broader applicability. Studies focused solely on insect consumption in children’s diets were excluded, as they fell outside the scope of this study.

In total, from the 76 publications analysed between 2012 and 2024, 54 studies were selected for analysis.

In addition to the scientific literature, relevant European and Portuguese legislation was consulted to support the topics addressed. This legislative review included 17 references.

## 3. Insects as Food

The practice of eating insects dates back to the hunter–gatherer phase of *Homo sapiens* and remains an integral part of many people’s diets today [3]. The earliest records of humans consuming insects trace back to the 8th century BC in the Middle East, where locust skewers were served at royal banquets [1,3,15]. Since then, numerous references to this practice have appeared in religious texts from Christianity, Judaism, and Islam, frequently mentioning locusts as food [1,15]. Throughout history, entomophagy has been documented in literary works, such as Aristotle’s *Historia Animalium*, which mentions the consumption of cicadas as a delicacy. The novel *Satyricon* by Gaius Petronius Arbiter describes a banquet scene in Ancient Roman society where the upper class consumed insects. Additionally, Li Shizhen’s in the Compendium of Materia Medica, a book on Chinese medicine from the Ming Dynasty, highlights the medicinal benefits of numerous insects [1,15]. Today, insect consumption differs significantly from the past. It is a practice deeply rooted in the culture and traditions of approximately two billion people across Africa, Asia, Latin America, and Oceania, where insects are valued as a nutritious and appetising food source [1]. However, in Western countries, entomophagy still faces significant cultural aversion and repulsion despite the increasing interest in its production, driven by its nutritional value and environmental benefits [1,15]. To counteract this stigma, and with the aid of technological advancements, efforts have been made to adapt the consumption of insects through innovative processing methods. Nowadays, insects are incorporated into various food products, such as protein bars, cookies, and burgers, appealing to modern tastes and dietary preferences [1,15].

The word “insect” originates from the Latin word *insectum*, meaning “cut or divided into sections” [1]. These invertebrates have a chitinous exoskeleton, with a body segmented into three tagmata (head, thorax, and abdomen), three pairs of jointed legs, compound eyes, and two antennae [1]. Insects belong to the kingdom Animalia, phylum Arthropoda, and class Insecta, which includes numerous orders. Regarding edible insects, 2205 species have been identified in 25 different orders [1,16]. Among these, Coleoptera is the most prevalent, constituting 32.0% of all edible insect species, followed by Hymenoptera (15.5%), Lepidoptera (15.2%), Orthoptera (14.1%), and Hemiptera (11.4%). Other orders, such as Isoptera, Odonata, Diptera, Dictyoptera, and Megaloptera, have lower representation [16].

## 4. Composition and Nutritional Value

The nutritional value of insects varies significantly, primarily due to the wide diversity of species [1,3]. This value also differs based on their rearing environment (natural habitat or captivity), sex, diet, and developmental stage [1,3,17]. As with most foods, the preparation and processing methods (e.g., drying, boiling, or frying) applied before consumption influence their nutritional composition [1]. However, insects are generally considered good sources of fats, proteins, fibre, vitamins, and minerals, making them an important food source for many communities worldwide [1,3].

**Figure 1:**
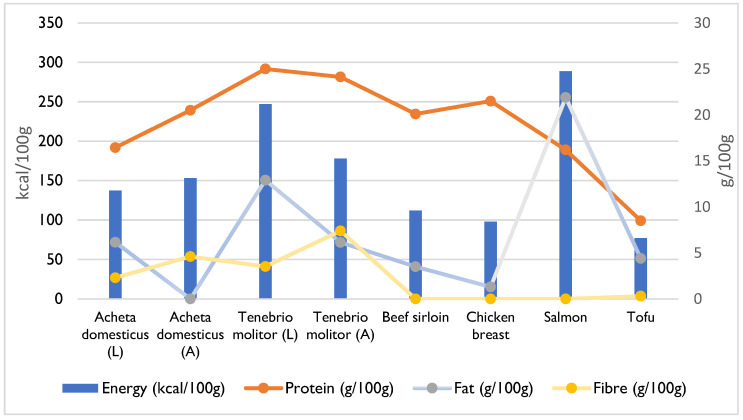
Comparison of food energy and the basic nutrient content of edible insects and traditional food sources, expressed per 100 g of edible portion [21,23]. A—adult insect; L—larval form.

The energy value of insects varies with the species and developmental stage and is closely related to their lipid content [3,10]. Typically, insect larvae are generally higher in calories than adult insects [10]. On average, the energy content of insects ranges from 200 kcal to 700 kcal per 100 g of dry weight (Figure 1) [10]. For instance, many species of flies (*Diptera* spp.) have low fat content, resulting in an energy value of around 200 kcal/100 g [10]. In contrast, some species in the order Lepidoptera exhibit high energy values, exceeding 700 kcal/100 g, such as the *Phasus triangularis* caterpillar, which has 776.9 kcal/100 g [10]. Generally, many edible insects, including crickets, locusts, and caterpillars, fall within the range of 400 kcal to 500 kcal/100 g [10].

Proteins are the main components of insects [3,17]. On average, the content of this macronutrient in edible insects varies between 35% and 60% of their dry weight or 10% and 25% of their fresh weight [11]. The protein content differs between and within the different orders that comprise the Insecta class; however, according to various studies, the order Orthoptera includes the insect species that are richest in protein [18]. For example, the species *Schistocerca gregaria* (Orthoptera) has protein content of 76% on a dry matter basis [19]. Within the same species, the protein content also varies according to the development stage of the insect: adults have the highest protein content, followed by larvae and finally pupae [10]. Insects from most taxonomic orders provide a complete profile of essential amino acids—comprising approximately 46% to 96% of their total amino acid content—along with various non-essential amino acids [10]. They are particularly rich in leucine, lysine, valine, threonine, phenylalanine, and histidine. Moreover, the biological value of insect-derived proteins is notably high, as their essential amino acid composition closely aligns with the proportions recommended for human nutrition. For example, the biological value of the species *Gryllus assimilis*, *Cirina forda*, *Melanoplus foedus*, and *Macrotermes nigeriensis* varies between 85% and 93%, exceeding the biological value of milk casein, which has a biological value of 73% [10,12]. However, with regard to protein digestibility, insects show very variable values due to the presence of the exoskeleton. This structure contains mainly chitin, which is difficult for humans to digest in large quantities. During processing, the exoskeleton of the insect is removed, and, consequently, some studies have shown that the insects’ digestibility increases to values between 77% and 98% [11]. Additionally, chitin can cause the overestimation of the protein content when total nitrogen methods such as Kjeldahl are employed, as these do not differentiate between protein and non-protein nitrogen sources. Consequently, it is recommended to apply species-specific nitrogen-to-protein conversion factors, which typically range from 4.76 to 5.60, instead of the conventional factor of 6.25. This approach provides a more accurate estimation of the true protein content in edible insects [17].

Lipids are the second most abundant macronutrients found in insects [3,12,19]. Their fat content typically ranges from less than 5% to over 50% of dry matter, depending on several factors [12,20]. The developmental stage of the insect is a predominant factor in fat analysis since, for most species, the levels are higher in the larval stage and lower in the adult stage [12,21]. For instance, *Tenebrio molitor* has fat content of 12.91% in the larval stage, which decreases to 6.14% in the adult stage, a reduction of approximately 50% [21]. Sex is also an important factor, as female insects have higher fat content than males [10]. The species, season, diet, and habitat must also be considered [3,10,12]. For example, insects in the order Coleoptera have higher fat content than other orders, with an average of 33.40%, while Orthoptera have only 13.41% [12]. The lipid composition of insects primarily consists of triglycerides, comprising a glycerol molecule and three fatty acids, accounting for about 80% of the total lipids. The remaining 20% includes phospholipids and sterols, such as cholesterol [3]. The fatty acid profile influences the nutritional quality of food fat—that is, the composition of saturated fatty acids, monounsaturated fatty acids, and polyunsaturated fatty acids determines the nutritional quality of insect fat [11]. The fatty acid profiles of insects vary with the species and diet; however, they are generally rich in essential unsaturated fatty acids, such as linoleic acid (an omega-6 fatty acid) and α-linolenic acid (an omega-3 fatty acid) [1,3,7]. However, despite their health benefits, unsaturated fatty acids will lead to the rapid oxidation of insect food products during processing, causing them to become rancid quickly [1].

The carbohydrate content of edible insects is mainly represented by fibre, which is present in considerable quantities [10]. The crude fibre content in insects ranges from 0% to 86%, with the highest levels being observed in the order Hemiptera, where the species *Aspongopus nepalensis* stands out, with 33.5% of fibre in relation to its dry weight [19,20]. The most common form of fibre in insects is chitin, an insoluble form of fibre derived from the exoskeleton [13]. Chitin is difficult for humans to digest due to the lack of the chitinase enzyme responsible for hydrolysing this polysaccharide. However, in countries where entomophagy is a common practice, greater activity of this enzyme has been recorded, in contrast to Western countries [1,12,13]. Generally, the chitin content of insects ranges from 2.7 mg/kg to 49.8 mg/kg (fresh weight) and from 11.6 mg/kg to 137.2 mg/kg (dry weight) [1,10,13].

Analysing the micronutrient content of edible insects, particularly vitamins, it is observed that many species are good sources of B vitamins [1]. Vitamin B1 (thiamine), a coenzyme in carbohydrate metabolism, is present in most species at concentrations ranging from 0.1 mg to 4 mg per 100 g of dry matter [1]. Vitamin B2 (riboflavin), with critical metabolic functions, is found in concentrations between 0.11 mg and 8.9 mg per 100 g of dry matter in most insects [1]. Notably, *Acheta domesticus* contains high levels of vitamin B12, with values of around 5.4 μg per 100 g of dry matter, distinguishing it from other species [10]. In contrast, the vitamin A (retinol and β-carotene) levels are significantly lower, indicating that insects are not optimal sources of this micronutrient; for example, *Tenebrio molitor* larvae have vitamin A levels below 20 µg per 100 g of dry matter [1]. Vitamin E (tocopherol) is present in some insects, such as ground and freeze-dried silkworm powder (*Bombyx mori*), which contains approximately 9.65 mg per 100 g [1]. Additionally, some studies have identified the presence of vitamins C, D, and K in edible insects, depending on the species [12]. However, according to some authors, studies on the vitamin content of edible insects are considered insufficient [20].

Insects are generally important sources of essential minerals, including calcium, copper, potassium, iron, phosphorus, manganese, magnesium, sodium, and zinc [3,10]. These micronutrients play a crucial role in the proper functioning of the human body, so inadequate intakes can lead, for example, to growth retardation, delayed sexual and bone maturation in cases of zinc deficiency, or the development of diseases such as anaemia in cases of iron deficiency [1]. These deficiencies are mostly observed in developing countries, where the inclusion of insects in the daily diets of their populations could be part of the solution to prevent these problems [1]. In this regard, the mopane caterpillar *Gonimbrasia belina* (Lepidoptera) stands out, as it contains iron levels ranging from 31 mg to 77 mg per 100 g of dry matter, exceeding those typically found in other widely consumed food sources, like beef, which contains 6 mg of iron per 100 g of dry matter [1,10,20]. Another example is the palm weevil larva *Rhynchophorus phoenicis* (Coleoptera), which contains 26.5 mg of zinc per 100 g of dry matter, more than double the concentration of 12.5 mg of zinc per 100 g of dry matter found in beef [1].

Given these properties, edible insects can also be used to produce supplements for athletes. A comparative analysis was conducted on the nutritional composition, micronutrient levels, amino acid profiles, and chemical scores of *Gryllodes sigillatus* (adult) protein in the form of whole flour, defatted flour, and protein isolate. These parameters were evaluated against widely used commercial protein supplements, namely whey protein concentrate and micellar casein [22]. In general, defatted cricket flour most closely resembled commercial supplements in nutritional value, containing 73.68% protein. It was also rich in essential minerals, including iron (4.59 mg/100 g dry weight), zinc (19.01 mg/100 g dry weight), and magnesium (89.74 mg/100 g dry weight) [22]. However, the amino acid composition of the protein preparation closely resembled that of commercial supplements, with total protein content of 694 mg/g [22]. Therefore, while crickets can be considered a natural and valuable protein source for the production of high-quality protein supplements, it is essential to emphasise that the nutritional value of insects can vary significantly due to various factors, including the insects’ batch [22]. To use insects for supplement production, the breeding conditions must be optimised to ensure consistent quality, which would streamline the production process. Additionally, further research is needed to evaluate the potential of insect protein in athletes’ nutrition [22].

Finally, it is possible to compare the nutritional compositions of various protein sources commonly consumed worldwide, such as beef sirloin, chicken breast, salmon, and tofu, with those of edible insects—specifically, *Acheta domesticus* and *Tenebrio molitor*, in both their larval and adult stages [21,23]. These two species are chosen as they are among the few authorised in the EU and have been the most extensively studied. It can be seen that, among these foods, the *Tenebrio molitor* species has high protein content in both stages of development, even exceeding the values recorded for beef and chicken (Figure 1) [21,23]. As already mentioned, the larvae of *Tenebrio molitor* have higher fat content than the adult insects, but the opposite is true for the *Acheta domesticus* species [21,23]. Consequently, the energy value of the *Tenebrio molitor* larvae is also higher, with values close to those of salmon, which also has high lipid content. Another difference can be seen in the fibre content, which is practically non-existent in meat, fish, and tofu, unlike in insects (Figure 1) [21,23].

**Figure 2:**
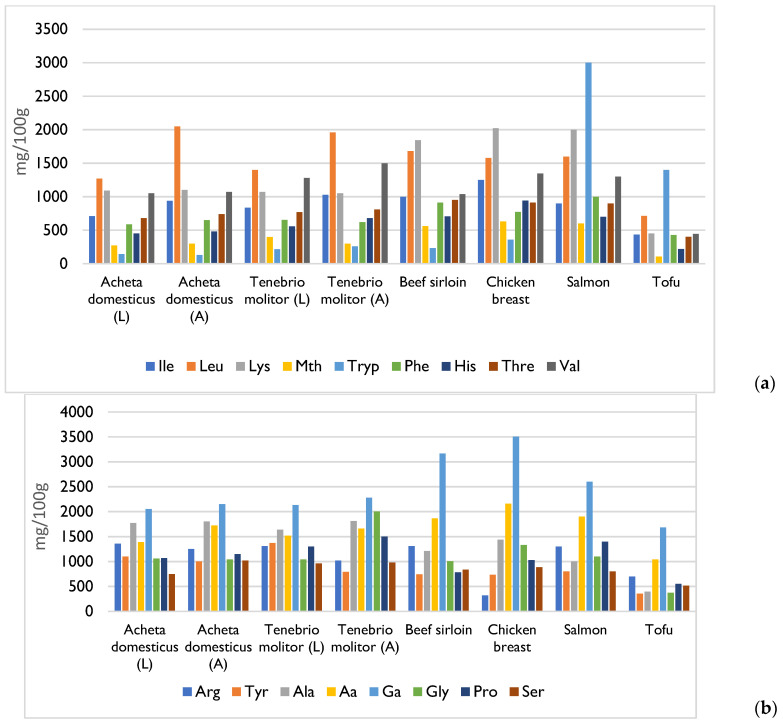
Comparison of the essential (**a**) and non-essential (**b**) amino acid compositions of edible insects and traditional food sources. Ile—isoleucine, Leu—leucine, Lys—lysine, Mth—methionine, Phe—phenylalanine, Tyr—tyrosine, Thre—threonine, Tryp—tryptophane, Val—valine, Arg—arginine, His—histidine, Ala—alanine, Aa—aspartic acid, Ga—glutamic acid, Gly—glycine, Pro—proline, Ser—serine, A—adult insect, L—larval form.

Figure 2 illustrates that both meat and edible insects serve as complete sources of animal protein, providing all essential amino acids. This is also true for salmon and tofu, although tofu has lower levels of these amino acids compared to the others [21,23].

**Figure 3:**
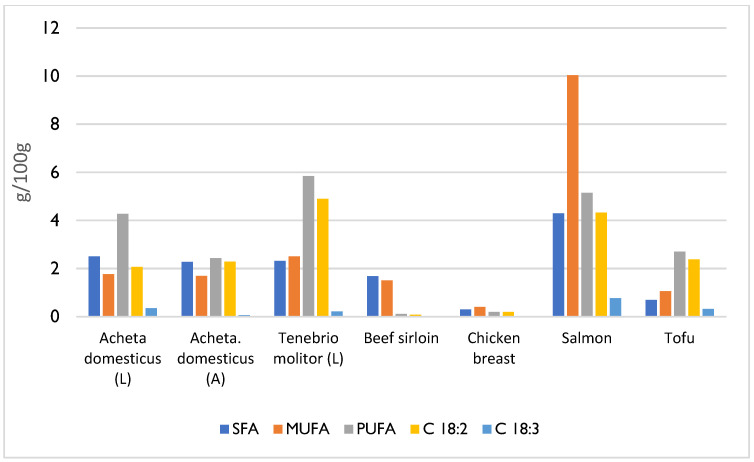
Comparison of the fatty acid compositions of edible insects and traditional food sources. SFA—saturated fatty acids, MUFA—monounsaturated fatty acids, PUFA—polyunsaturated fatty acids, C 18:2—linoleic acid, C 18:3—α-linolenic acid, A—adult insect, L—larval form.

The presence of linoleic acid, one of the essential fatty acids, is particularly important. As shown in Figure 3, the linoleic acid content was much higher in insect species than in meat sources. However, salmon remains the food richest in fatty acids [21,23].

**Figure 4:**
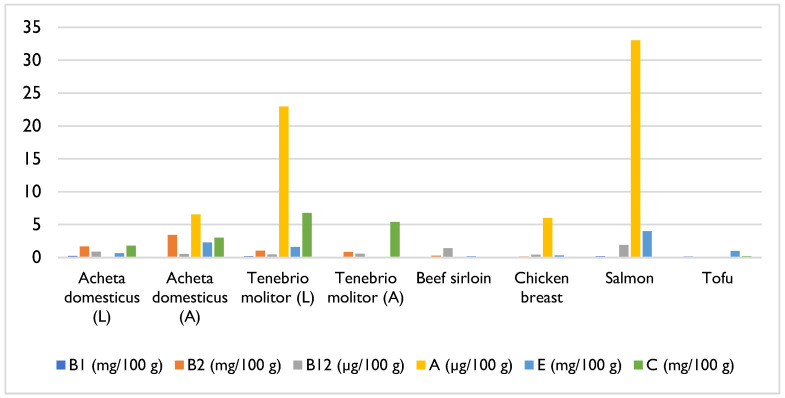
Comparison of the vitamin content of edible insects and traditional food sources. B1—thiamine, B2—riboflavin, B12—cobalamin, A—vitamin A, E—vitamin E, C—vitamin C, A—adult insect, L—larval form.

The vitamin content varies significantly between insects and traditional protein sources. Insects, across different species and developmental stages, consistently showed higher levels of vitamin E, riboflavin, and vitamin C compared to meat. Salmon, on the other hand, had the highest levels of cobalamin, a vitamin not found in non-animal products (Figure 4) [21,23].

**Figure 5:**
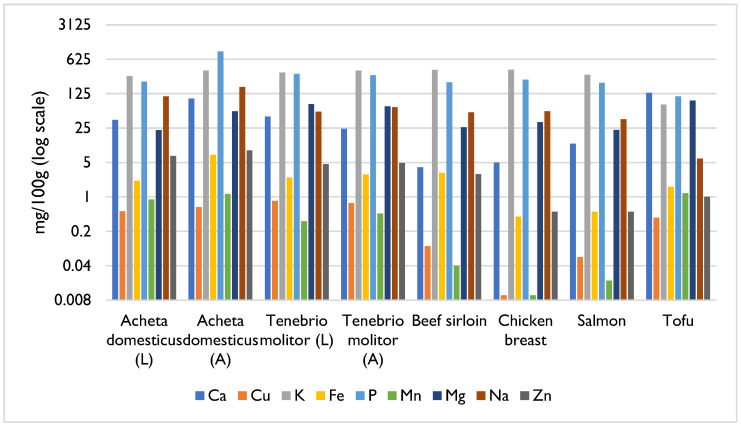
Comparison of the mineral content of edible insects and traditional food sources. Ca—calcium, Cu—copper, K—potassium, Fe—iron, P—phosphorus, Mn—manganese, Mg—magnesium, Na—sodium, Zn—zinc, A—adult insect, L—larval form.

As illustrated in Figure 5, both meat and insects are important dietary sources of minerals. However, insects—irrespective of the species or developmental stage—generally contain higher concentrations of calcium, zinc, copper, and manganese compared to meat (Figure 5) [21]. Regarding iron, meat is recognised as a source of bioavailable haem iron, primarily derived from haemoglobin and myoglobin. For example, beef sirloin contains more iron (3.1 mg/100 g) than poultry (0.4 mg/100 g). Insects, in turn, although presenting iron levels comparable to those of meat, contain this mineral predominantly in its non-haem form, which is generally less efficiently absorbed by humans [17,21,24]. Tofu also has high levels of calcium, manganese, and magnesium (Figure 5) [21,23].

## 5. Entomophagy Benefits

### 5.1. Health Benefits

Edible insects contain bioactive compounds that exhibit a wide range of beneficial bioactivity, including antioxidant, antihypertensive, and anti-inflammatory effects. These properties can have a positive impact on human health [25].

#### 5.1.1. Antioxidant Activity

Reactive oxygen species—including superoxide anion (O_2^−^_), hydrogen peroxide (H_2_O_2_), and hydroxyl radicals (•OH)—are byproducts of normal cellular metabolism. When present in excess, these molecules can inflict considerable cellular damage. Persistent oxidative stress is associated with the onset and progression of various pathologies, including cancer, cardiovascular diseases, and arthritis [11].

The antioxidant activity of protein hydrolysates from *Blaptica dubia*, *Gromphadorhina portentosa*, *Locusta migratoria*, *Zophobas morio*, and *Amphiacusta annulipes* was evaluated in a study using various methods, including free radical scavenging activity, ion-chelating activity, and reducing power assays [26].

In general terms, the first method determines the antioxidant potential of compounds based on their ability to scavenge free radicals such as the stable 2,2-diphenyl-1-picrylhydrazyl radical (DPPH^●^) or the 2,2-azinobis (3-ethylbenzthiazoline-6-sulfonic acid) radical (ABTS^●+^) [27]. At the end of the experiment, the IC_50_ values provide a quantitative measure of the radical scavenging affinity [27]. The results for this method showed that *Amphiacusta annulipes* hydrolysates had the highest antiradical activity against DPPH^●^ (IC_50_ = 19.1 µg/mL), while *Zophobas morio* had the highest antiradical activity (4.6 µg/mL) against ABTS^●+^ [26,28].

The second method measures ion chelating activity—that is, the binding affinity between a reagent and a metal ion, such as Fe^2+^ [29]. In this case, *Amphiacusta annulipes* peptides demonstrated the highest Fe^2+^ chelation ability, with a value of 58.8% [26,28].

The reducing power assay, as the name suggests, evaluates the reducing power of compounds by converting Fe^3+^ to Fe^2+^, which can be a significant indicator of their potential antioxidant activity [30]. *Amphiacusta annulipes* peptides demonstrated reducing power of 0.652 [26,28].

#### 5.1.2. Hypotensive Effects

Hypertension is currently recognised as a major public health problem [26]. In this context, a study investigated the inhibition of enzymes associated with the development of metabolic syndrome, such as angiotensin-converting enzyme (ACE), using peptide fractions derived from the simulated gastrointestinal digestion and absorption of heat-treated edible insects (*Tenebrio molitor*, *Schistocerca gregaria*, and *Gryllodes sigillatus*) [31]. Following the in vitro absorption process, all hydrolysates exhibited high inhibitory activity [31]. Furthermore, the thermal treatment of the insects enhanced their ACE-inhibitory activity [31]. The greatest ACE inhibition was observed for the hydrolysate from the baked *Gryllodes sigillatus* (IC_50_ = 28.2 μg/mL) [31]. However, the best results were found for peptide fractions obtained from boiled *Schistocerca gregaria* (IC_50_ = 3.95 μg/mL) [31]. Generally, divided peptide fractions derived from insect hydrolysates show higher inhibitory activity than hydrolysates obtained through the simulated absorption process, with some exceptions, such as the ACE-inhibitory effects of peptide fractions from *Gryllodes sigillatus* [31]. This suggests that the enhanced activity may result from the synergistic effects of the peptides present in the hydrolysate [31].

#### 5.1.3. Anti-Inflammatory Activity

A study investigated the effects of heat treatment on three insect species—*Tenebrio molitor*, *Schistocerca gregaria*, and *Gryllodes sigillatus*—focusing on the antioxidant and anti-inflammatory activity of peptides obtained through in vitro gastrointestinal digestion and absorption [32]. The anti-inflammatory activity was expressed as lipoxygenase (LOX)- and cyclooxygenase-2 (COX-2)-inhibitory activity [32]. The *Schistocerca gregaria* protein hydrolysate was the most effective inhibitor of LOX (IC_50_ value 0.65 mg/mL), while the raw *Schistocerca gregaria* hydrolysate inhibited COX-2 most efficiently (IC_50_ value 10.91 μg/mL) [28,32]. The peptide fraction from the *Schistocerca gregaria* protein hydrolysate showed the best LOX- and COX-2-inhibitory activity among the peptide fractions (IC_50_ values 3.13 μg/mL and 5.05 μg/mL, respectively) [32]. Generally, the best LOX- and COX-inhibitory activity was determined for the peptide fractions and hydrolysates obtained after the digestion of the insect protein preparations [32]. Better inhibitory activity was noted for the peptide fractions than for the hydrolysates [32]. The conducted research proved that the activity of LOX and COX-2 was effectively inhibited by the edible insect hydrolysates and peptide fractions [32].

### 5.2. Environmental Benefits

Raising insects for human consumption provides numerous environmental benefits. Firstly, it is crucial to emphasise the high food conversion efficiency of insects, as measured by the feed conversion ratio (FCR) [5,7,8,9]. The FCR is a commonly used indicator to assess the efficiency of livestock production. It represents the ratio of feed consumed to animal weight gain. Thus, low FCR values indicate high feed conversion efficiency [33]. Edible insects have an average FCR of about 1.7, while the FCR for beef, pork, and chicken is around 10, 5, and 2.5, respectively [5,8,9]. This difference occurs because insects are poikilothermic animals, meaning that their internal temperature varies considerably. Therefore, they do not need food to maintain a constant body temperature [1,5].

Insect farming is also associated with significantly lower greenhouse gas emissions than livestock production [3]. In this context, species such as *Acheta domesticus*, *Blaptica dubia*, *Locusta migratoria*, and *Tenebrio molitor* show methane production values ranging from 0.00 to 0.16 g/kg of body mass per day, in sharp contrast to the 0.239 g/kg of body mass per day produced by cattle. For ammonia production, the trend observed continues, with species like *Acheta domesticus* and *Blaptica dubia* showing values of around 3.0–5.4 mg/kg of body mass per day. In comparison, pigs exhibit values ranging from 4.8 to 75 mg/kg of body mass per day and cattle from 14 to 170 mg/kg of body mass per day [9].

In terms of water consumption, although specific values for most edible insect species are not well documented, it is generally estimated that their water use is lower compared to livestock farming [1]. Insects can often meet their water needs from their feed or substrates. For example, species such as *Tenebrio molitor* are known for their resistance to water scarcity [5,7,9]. In contrast, the production of 1 kg of chicken meat requires 2300 L of water, while the same amount of pork requires 3500 L and beef up to 22,000 L, with some beef production methods requiring as much as 43,000 L [1,3].

Finally, it is also important to highlight the reduced land area required for rearing insects [3]. In comparative terms, producing 1 kg of *Tenebrio molitor* requires 18 m^2^, whereas the same amounts of chicken, pork, and beef require progressively larger areas: 51 m^2^, 63 m^2^, and 254 m^2^, respectively [3].

### 5.3. Socioeconomic Benefits

In developing countries, where some local cultures commonly practice entomophagy, cultivating, capturing, preparing, and selling insects can provide various subsistence opportunities for these communities [14,34]. This particularly benefits the most disadvantaged populational groups, such as women, the elderly, landless dwellers, and children. Insects can be collected directly from the environment, without complex technical equipment or significant investments in basic breeding and collection tools [14]. Furthermore, insect consumption is associated with improvements in the nutritional quality of these people’s diets [14,34]. For example, diets primarily based on tubers in Angola and Papua New Guinea result in deficient intake of lysine and leucine. In these regions, the inclusion of the termite *Macrotermes subhyalinus* and beetle larvae from the *Rhynchophorus* family can help to address these deficiencies. The incorporation of these insects into the diet, when combined with traditional tubers rich in tryptophan and aromatic amino acids—nutrients that are present at lower levels in these insects—can substantially improve the overall nutritional adequacy [10]. Finally, these practices can also generate financial returns through selling surplus insects. For instance, in countries like Thailand, profits from insect sales often exceed those from conventional animal protein sources, such as cattle, pork, and poultry [1].

In developed countries, breeding, collecting, and processing insects can create business opportunities [18]. They can be used to develop insect-based food products for human consumption [18]. As the awareness of the nutritional benefits of insects grows, more consumers are open to including them in their diets [18]. This creates a lucrative business market for the innovation and development of insect-based foods that meet evolving consumer preferences.

## 6. Legal and Regulatory Framework

The breeding, commercialisation, and use of insects for human consumption have recently been authorised in the European Union (EU) [3]. These are regulated following legislation relating to novel foods, which was first introduced via European Commission Regulation (EC) 258/97 [35]. According to this regulation, a novel food is defined as food that has not been consumed to a significant degree by humans in the EU before 15 May 1997 [36]. It also covers newly developed, innovative food and food produced using new technologies and production processes, as well as food that is or has been traditionally consumed outside the EU [36].

As a result of scientific and technological advances, Regulation (EC) 258/97 was repealed and replaced with European Union Regulation (EU) 2015/2283, which is currently in force [37]. This regulation revised, clarified, and updated the categories of novel foods, which now include whole insects and their parts [37]. The authorisation procedure for marketing new foods and traditional foods from third countries in the EU has also been simplified [36,38]. Edible insects can now be legally placed on the EU market, but marketing authorisations are required in advance [36,37,38]. These authorisations are granted following the submission of an application to the EC, a safety assessment by the European Food Safety Authority (EFSA), and, where applicable, favourable votes from EU Member States [36,37,38].

Once the EC grants authorisation and the EFSA provides a positive opinion, these foods must be included in the “EU List of Novel Foods” to be placed on the market [36,37,38]. This list, defined by Regulation (EU) 2017/2470, contains all novel foods and traditional foods from third countries currently authorised in the EU [39]. The list details the name, conditions of use, labelling, and other requirements, as well as specifications of the novel foods and traditional foods from third countries [36,38,39]. It is updated whenever a food or ingredient is authorised as a novel food or when a traditional food from a third country is successfully notified [36,38,39].

Following the entry into force of Regulation (EU) 2015/2283, Implementing Regulation (EU) 2017/2469 and Implementing Regulation (EU) 2017/2468 also became applicable [40,41]. These regulations establish requirements for administrative and scientific data concerning applications to place novel foods or traditional foods from third countries on the Union market [40,41]. Traditional foods from third countries are defined as foods that have been consumed as part of the regular diet of a significant number of people for at least 25 years in at least one non-EU country [36,38].

Regarding labelling, novel foods must comply with the general requirements outlined in Regulation (EU) 1169/2011 [42]. Additionally, any claims made about potential nutritional or health benefits must comply with Regulation (EC) 1924/2006 [43].

It is worth noting that imports from third countries are allowed, but only from countries specified in Implementing Regulation (EU) 2021/405 [43]. This is subject to the products being duly authorised and the subsequent update of the Union List of Novel Authorised Foods [43]. Currently, the regulation lists Canada, Switzerland, South Korea, Thailand, and Vietnam as authorised third countries for the importing of insect shipments into the EU [43].

The insect species currently authorised for commercialisation are *Acheta domesticus* (frozen, dried, powder), *Alphitobius diaperinus* (frozen, paste, dried, and powdered larva forms), *Acheta domesticus* (partially defatted powder), *Locusta migratoria* (frozen, dried, powder), *Tenebrio molitor* (dried larvae), and *Tenebrio molitor* (frozen, dried, and powdered larva forms) [36,38]. These authorisations are detailed in Implementing Regulation (EU) 2022/188, Implementing Regulation (EU) 2023/58, Implementing Regulation (EU) 2023/5, Implementing Regulation (EU) 2021/1975, Implementing Regulation (EU) 2021/882, and Implementing Regulation (EU) 2022/169, respectively [44,45,46,47,48,49]. It should be noted that the rearing and commercialisation of all authorised edible insect species are exclusively permitted for applicants who have successfully completed the novel food authorisation process.

However, other insect species may be traded under the Transitional Measures [36,38]. Prior to 1 January 2018, under Regulation (EC) 258/97 (now repealed), there was ambiguity regarding whether whole insects qualified as novel foods [35,36,38]. Most EU countries interpreted the legislation to classify whole insects as novel foods, requiring their authorisation before being placed on the market [36,38]. Conversely, countries such as Finland, Denmark, and The Netherlands interpreted the legislation differently, allowing the commercialisation of whole insects without considering them novel foods [36,38]. This discrepancy led to a legal challenge before the European Court of Justice in October 2020, which concluded that whole insects were not covered by Regulation (EC) 258/97 [36,38]. Consequently, during a transitional period, whole insects can be placed on the EU market, since the provided specific conditions are met [36,38]. This permission is based on transitional provisions of the new legislation, allowing foods marketed under previous regulations to continue to be sold while awaiting authorisation under Regulation (EU) 2283/2015 [36,38]. However, there are two conditions for the commercialisation of insects during this transitional period:

1. They must have been legally placed on the market in an EU country before 1 January 2018 [36,38];
2. An application for commercialisation authorisation must have been submitted for the insect as a novel food or traditional food from a third country before 1 January 2019 [36,38].

It is important to note that insects can only be sold or used whole (not alive) and ground (e.g., as flour) [36,38]. The sale of insect parts or extracts thereof is not permitted [36,38].

In the U.S., edible insects are regulated by the Food and Drug Administration (FDA) under the Food, Drug, and Cosmetic Act. They must meet hygiene, safety, and labelling standards and be produced under Good Manufacturing Practices. Wild-harvested insects are not allowed. Insects used as ingredients may require food additive approval, unless considered Generally Recognised As Safe (GRAS). GRAS and the Food Additive Petition (FAP) are parallel regulatory pathways, both requiring safety evidence and applying to specific uses [50,51].

In Canada, edible insects with a history of safe consumption may be classified as traditional foods and are exempt from pre-market approval. Species like *Tenebrio molitor* and *Gryllodes sigillatus* fall under this category, while others, such as grasshoppers or *Hermetia illucens*, require full safety assessment. The Canadian Food Inspection Agency (CFIA) enforces food safety and hygiene standards, and provincial authorities regulate production practices [50,51].

In Asia, edible insect regulation varies widely across countries. Thailand has established regulatory frameworks primarily for cricket farming through Good Agricultural Practices, ensuring product safety and quality. However, specific regulations for the farming, processing, and use of other edible insects are still under development. The Thai Food and Drug Administration oversees food safety broadly [50]. Most African countries lack specific regulations for edible insects. In South Africa, the regulatory framework is fragmented, with multiple government bodies overseeing food and feed safety but no specific laws for insect farming or insect-based foods. Insects are not considered novel foods [50].

## 7. Food Safety

### 7.1. Insect Farming

In developing countries, there is traditional and cultural knowledge regarding the consumption of edible insects as food, with production being predominantly household and small-scale [1,2]. In developed countries, the lack of processing technology reflects the fact that edible insects are not recognised as food or feed sources. For insects to be economically viable in the food and feed industries, significant quantities of high-quality insects must be consistently produced. Achieving this requires the automation of both farming and processing methods, a challenge that must be addressed for the sector to develop effectively [1,2,52].

The large-scale production of edible insects involves addressing several important aspects [1]. Firstly, the processing methods must be equally controlled [1]. Regarding legislation, regulations and guidelines for producers have to be developed, covering areas such as feed sourcing and standards, animal welfare, biosecurity to prevent escapees, and disease management [1]. Additionally, the final product should be easy to transport and store, with a preferably long shelf life. During processing, it is crucial to maintain or improve the nutritional quality of the insects. Finally, the product must be competitive with existing market alternatives [1]. The establishment of farms processing insects for food and feed will likely become a global reality, driven by the growing demand for sustainable food and feed sources.

The industrial insect production system revolves around managing the life cycle of the target species to be produced. Similarly to other livestock production methods, this system is divided into stages, including reproduction, rearing, and fattening (production) phases [52,53].

The life cycle of *Tenebrio molitor* comprises four distinct stages: egg, larva, pupa, and adult [53]. The duration of each stage, and thus the total length of the life cycle, which ranges from 280 to 630 days, depends on environmental factors such as the temperature, relative humidity, and availability of food and water [53]. Thus, production is carried out according to the different phases of the life cycle, resulting in the production unit being divided into reproduction, incubation, and production/bioconversion areas [53].

The breeding area can consist of boxes containing adults, provided with a solid substrate and a moisture source, such as vegetables or other water supplies. The boxes with adults may have fine mesh bottoms or other systems for autonomous egg separation, or they can use a substrate that is regularly replaced through sieving. In both cases, the eggs produced are incubated under conditions suitable for larval hatching, with food provided in appropriate quantities and favourable environmental conditions maintained [53]. After several weeks of development, the larvae are separated and either selected for reproduction, allowing them to complete their life cycle, or sent to production. In batches chosen for reproduction, the larvae may reach the pupal stage. However, this stage is susceptible to predation by other individuals, necessitating careful separation of the different stages as they are obtained [53].

The larvae obtained in the previous step can be kept in the original substrate or transferred to new substrates [53]. If vegetable byproducts are to be used, the larvae can be introduced to them at this stage, taking into account the relative humidity of these byproducts. Most producers use substrates specifically formulated for this purpose, such as compound feed or simple mixtures of cereals or bran [53]. Mealworm larvae can be reared in stackable boxes, generally sized and weighted for easy individual handling, with side walls designed to prevent escapes. It is recommended that rooms containing these boxes be organised with systems to ensure traceability between production batches and to maintain physical separation between the different stages of production [53]. At the end of the production/bioconversion period, which varies based on the substrate used and the environmental conditions, insects are separated while still in the larval stage and sent for processing. Substrate bioconversion results in a fine particulate fertiliser that easily forms dust [53]. Workers responsible for separating or handling these insects should use protective equipment to safeguard their respiratory tracts. To prevent possible environmental contamination, it is recommended that the fertiliser undergoes biosafety procedures to eliminate any remaining larvae before distribution. This may involve the use of chemical or physical methods to eliminate all insect forms, including eggs, larvae, or adults [53].

*Regarding Acheta domesticus*, this species follows a three-stage life cycle—egg, nymph, and adult—which is completed within 2 to 3 months under optimal temperatures (26–32 °C). Eggs are deposited in moist substrates and hatch after approximately 14 days. Nymphs resemble adults but lack wings and, in females, the ovipositor; they undergo 8 to 10 molts before reaching maturity. In the adult stage, individuals focus on feeding and reproduction, with females capable of laying up to 200 eggs during their lifespan [53].

In industrial settings, *Acheta domesticus* females oviposit in humid horticultural substrate, reused across several cycles unless contaminated by fungi. A mesh prevents adults from ingesting the substrate. Near hatching, substrates are placed in nursery units and newly emerged nymphs fall into clean chambers, allowing efficient collection and allocation to rearing units [53].

Cohorts are managed with strict age separation (typically ≤48 h) and full traceability. Crickets are reared in climate-controlled, ventilated enclosures with cardboard shelters to increase the surface area and stimulate natural behaviour. Feeding is plant-based and dry, with water supplied via either flowing or static systems, requiring routine sanitation. All materials used must prevent ingestion or injury, and shelter materials are replaced after around five cycles. Durable, washable alternatives (e.g., rigid PVC) are being considered for future use [53].

To ensure food safety, insect production must adhere to strict hygiene standards and comply with relevant food regulations, thereby guaranteeing the safety of products intended for human and animal consumption [1,2,52].

### 7.2. Preservation and Storage

Like various meat products, insects are rich in nutrients and moisture, providing a favourable environment for microbial survival and growth [1].

Edible insects can be farmed; however, they are typically collected from the wild. As a result, some species are only available seasonally, so they must be preserved and stored before processing or consumption [1,54]. The most common preservation methods are refrigeration, drying, acidification with vinegar, and lactic fermentation (the low pH prevents the growth of harmful microorganisms) [54,55]. Drying methods reduce the total water content and, therefore, its availability for degradative reactions [54,55]. These include sun-drying, a traditional and low-energy drying method commonly used at the household level for preserving edible insects. It helps to prevent some microbial contamination and reduces or removes harmful compounds like neurotoxins. However, sun-drying has significant drawbacks, including the poor hygienic quality of the process and the final product. Insects are considered dehydrated based on appearance alone, without measuring the moisture content or water activity. Due to limited hygiene practices, dried insects can become contaminated through contact with soil and air [54,55,56]. In turn, freeze-drying uses low temperatures and water sublimation to minimise microbiological and oxidative degradation, resulting in a high-quality product with excellent nutritional value and a long shelf life. However, it is costly to scale up to industrial levels, and high-fat products can suffer lipid oxidation, which reduces protein solubility [54,55]. Oven-drying, while comparable to freeze-drying, is more cost-effective, reduces lipid oxidation, and maintains high protein solubility. The choice of drying technology impacts quality parameters beyond just water reduction, affecting protein functionality, lipid oxidation, and the colour. Therefore, the selection of the appropriate drying method should consider the intended use of the insect and the final form in which it will be consumed (whole, powdered, or sole ingredient) [54,55].

Traditional processing methods such as boiling, roasting, and frying are also commonly used for edible insects to ensure food safety and enhance their taste and palatability. Additionally, cultural preferences and sensory aspects (organoleptic properties) play significant roles in determining the preferred preservation methods [1,55].

Storage requirements vary based on the insect species and the form in which the product is marketed—for instance, as whole ready-to-eat insects or as a powder derived from dried material. For fresh insect products, freezing at −20 °C is preferred over refrigeration at 5–7 °C to better preserve microbial quality [54,55]. For dried and powdered edible insects, refrigeration is the most effective method to prevent oxidative and microbiological degradation. Additionally, combining refrigeration with vacuum or modified atmospheres significantly extends the shelf life of these products [54,55].

### 7.3. Biological Hazards

While entomopathogenic microbes are generally harmless to humans and animals, insects can still transmit harmful microorganisms, especially in less hygienic conditions [2,20]. Although the risk of zoonotic infections from edible insects appears to be low, more research is needed to clarify the potential risks for food and feed [2,20]. When considering potential biological hazards in insects, there are two types of microbiota to be aware of: those intrinsically associated with the insects’ lifestyles and those introduced during farming and processing [20].

#### 7.3.1. Bacteria

Numerous bacterial species have been associated with edible insects, whether farm-reared or wild-caught. These include species from the genera *Staphylococcus*, *Streptococcus*, *Bacillus*, *Pseudomonas*, *Micrococcus*, *Lactobacillus*, *Erwinia*, *Clostridium*, and *Acinetobacter* and members of the family *Enterobacteriaceae* [20]. Some of these genera and family members are pathogenic and opportunistic bacteria and contribute to the shortened shelf lives of edible insects [2]. To minimise the transmission of foodborne pathogens to humans through insect consumption, strict biosecurity measures should be implemented on insect farms to prevent contact with livestock animals [20]. *Campylobacter* and *Salmonella* have been isolated from insects close to affected livestock. Additionally, feeding experiments with houseflies (*Musca domestica*) using *Escherichia coli* O157:H7 have demonstrated that ingested bacteria can persist in the gut, crop, and mouthparts of the insect and can be excreted for up to three days post-feeding. This highlights the potential for houseflies to act as vectors in spreading bacteria [2,20]. The contamination of edible insects after processing is also a significant concern. For example, insects dried under the sun in humid environments may be vulnerable to microbial growth due to moisture [55]. Air-drying methods, especially when insects come into contact with soil, also present potential food safety challenges [55]. In many regions worldwide, “ready-to-eat” insects are typically roasted or fried—procedures known to eliminate foodborne pathogens effectively [55]. However, there is a risk of re-contamination or cross-contamination if these insects are not handled and stored hygienically before consumption [2].

#### 7.3.2. Viruses

Various virus species have been identified in edible insects for mass production systems [2]. These viruses pose potential risks to mass insect-rearing operations, potentially resulting in acute high mortality rates, significant growth declines during juvenile stages, and reduced reproductive performance in adults. Concerning viruses originating from vertebrates, they can persist in substrates and be acquired by insects used in food or feed production, raising transmission concerns [57]. This risk can be mitigated by careful substrate selection and effective processing methods [20,57].

Available data suggest that pathogenic viruses found in insects produced for food and feed are typically species-specific and are not considered dangerous to vertebrate animals and humans [20,57]. However, further research is needed to better understand the potential occurrence and transmission of arthropod-borne arboviruses through edible insects [2,20,57].

#### 7.3.3. Fungi

Insects can act as carriers of fungi and yeasts, posing a potential risk to animals and humans [2,20]. Significant amounts of yeast and fungi have been found in fresh, freeze-dried, and frozen insect species, such as *Tenebrio molitor* and *Locusta migratoria* [20]. From the species *Gonimbrasia belina*, dried under laboratory conditions, various fungi, including *Aspergillus* spp. and *Penicillium* spp., were isolated, some of which were mycotoxigenic [20]. Overall, any risk from fungi associated with insects produced for food and feed, or introduced during farming, processing, and storage, can be mitigated through stringent hygienic measures throughout the entire production chain [2,20].

#### 7.3.4. Parasites

The risk of the transmission of parasitic diseases to humans is higher from wild-harvested insects than from farmed insects [20]. This is due to the lack of confinement and controlled feeding practices in wild environments, unlike the regulated conditions in insect farming [20]. Investigations of certain insect species and human autopsies from regions where entomophagy is common indicate a possible risk of parasite transmission through insect consumption. For example, the zoonotic parasite *Dicrocoelium dendriticum* may be transmitted via edible ants [2,14,20]. Moreover, food- and waterborne protozoan parasites such as *Entamoeba histolytica*, *Giardia lamblia*, and *Toxoplasma* spp. have been detected in cockroaches [2,20]. Although parasites have been documented in wild insects and linked to occasional human infections, there is currently no evidence of such organisms in farmed insects. This absence is likely due to the controlled conditions of insect farming systems, which typically prevent the presence of intermediate hosts required for parasite development. Moreover, proper management practices, such as freezing and cooking, can mitigate potential risks before consumption [20].

### 7.4. Chemical Hazards

#### 7.4.1. Heavy Metals

Their accumulation in insects depends on the type of heavy metal, insect species, growth stage, substrate, and possibly the substrate’s packaging material [2,20,58]. The bioaccumulation factor (BAF) measures the accumulation of a chemical substance in an organism relative to its concentration in the surrounding environment, such as the food or water to which it is exposed [58,59]. It is calculated as the ratio of the substance’s concentration in the organism to its concentration in the environment [58,59]. For example, the BAF of lead in *Tenebrio molitor* was 34 when fed a diet of 100% organic wheat flour, compared to a BAF of 6.1 when fed a diet consisting of 75% organic wheat meal and 25% organic olive pomace. In addition, *Hermetia illucens* had a BAF of 20.4 for cadmium when fed a vegetable-based substrate in a carton. However, the BAF for cadmium dropped to 7 when the same vegetable-based substrate was provided in a plastic container [58]. Furthermore, using spiked feeding trials, black soldier flies were found to excrete most of the arsenic that they consumed. In contrast, yellow mealworm larvae were shown to accumulate arsenic, raising safety concerns [58].

#### 7.4.2. Mycotoxins

Several mycotoxins have been detected in edible insects, but they are typically found at levels that do not pose public health concerns. Tolerance to these contaminants varies depending on the insect species and the specific types of mycotoxins involved [2,20]. For instance, *Hermetia illucens* fed poultry feed spiked with aflatoxin B_1_ did not accumulate the toxin, with the levels in the larvae remaining below the detection limit of the analytical method used (0.10 μg/kg). In contrast, *Tenebrio molitor* larvae fed the same spiked substrate contained up to 1.44 μg/kg of aflatoxin B_1_—approximately 10% of the EU’s legal limit for feed materials [58]. In general, based on the studies conducted so far, the accumulation of mycotoxins in insects is not anticipated. They are rarely found in the insects’ bodies but are regularly detected in their excreted waste [2,20]. This suggests that the biotransformation of mycotoxins occurs, leading to varying levels of metabolites. For example, in a study where *Tenebrio molitor* was fed 79.9 μg/kg zearalenone (ZEN), the residual material contained 26.2 μg/kg ZEN, 6.8 μg/kg α-Zearalenol (ZEL), and 17.3 μg/kg β-ZEL [58]. In this context, further research is needed to better understand the metabolic pathways utilised by different insect species, the metabolites produced, and their potential toxicological effects on human and animal health [2,58].

#### 7.4.3. Veterinary Drugs

Insects reared on manure may be exposed to residues of veterinary drugs [20,58]. This risk is among the main reasons that the use of manure as a substrate for insect farming intended for human consumption is explicitly prohibited in the EU and effectively restricted in most other countries due to food safety regulations [50,60,61,62]. These medications may also be administered during rearing to prevent infections; however, they may hinder the insects’ development and contribute to the spread of antibiotic-resistant pathogens, potentially nullifying their intended benefits. Balancing the use of veterinary drugs during insect rearing represents a challenge, as it aims to control microbial growth while ensuring optimal insect growth and survival [20,58]. For instance, a study has shown that the antibiotic sulfonamide can impact the growth of *Hermetia illucens* [63]. This drug was added to the substrate at concentrations of 0, 0.1, 1, and 10 mg/kg [63]. Only the highest concentration significantly affected larval survival, reducing it to 30%. Additionally, it influenced the body weight and developmental progression of the larvae. Nevertheless, sulfamonomethoxine, sulfamethoxazole, and sulfamethazine were not detected in the prepupae, whereas sulfadiazine was found at concentrations of 1 and 10 mg/kg (0.5–0.8 mg per 100 prepupae) [63]. This indicates that *Hermetia illucens* larvae can reduce certain veterinary drugs from manure, thereby playing a role in environmental remediation efforts [63].

#### 7.4.4. Pesticides

Substrates used for rearing insects may contain pesticide residues [20]. One investigation assessed the presence of 393 pesticide residues in farmed insect species, including houseflies, bluebottle flies, blowflies, and black soldier flies, with detection limits between 10 and 50 μg/kg [20]. Notably, chlorpyrifos was identified at a concentration of 800 μg/kg (dry weight) in a sample of housefly larvae reared on milk powder and sugar in Wuhan, China. Similarly, piperonyl butoxide was detected at 200 μg/kg (dry weight) in a bluebottle fly sample from the UK. All remaining pesticide levels in the analysed insects were below their respective detection thresholds [58]. In terms of feed safety, the Codex Alimentarius sets maximum recommended concentrations for chlorpyrifos and piperonyl butoxide in animal feeds—such as alfalfa and pea fodder—at 5000 μg/kg and 2000 μg/kg, respectively. The limited data suggest that the insect feed contaminant levels are below these recommended maximum concentrations [58].

### 7.5. Allergens

Like other protein-rich foods, edible insects can trigger immunoglobulin E (IgE)-mediated allergic reactions in sensitive humans [1]. These adverse immunological reactions can be caused by injection, inhalation, skin contact, or the ingestion of the allergen. People who are regularly exposed to edible insects are one of the main groups at risk of developing these allergic reactions [20]. Conversely, they can also result from cross-reactivity when IgE antibodies, initially produced against one allergen, bind to another structurally related allergen. This phenomenon is common among allergens from taxonomically related species, such as crustaceans or house dust mites, due to pan-allergens—proteins that are highly conserved through evolution and capable of inducing allergic responses across related species [64]. Among the identified cross-reactive allergens, proteins such as α-actin, arginine kinase, enolase, fructose-1,6-bisphosphate aldolase, glyceraldehyde-3-phosphate dehydrogenase, and tropomyosin are frequently recognised as IgE-binding components [65]. In addition, other commonly occurring proteins—including α-amylase, glutathione S-transferase, myosin, paramyosin, triose-phosphate isomerase, and troponin—have also been reported as IgE-binding cross-reactive allergens across insects, crustaceans, and molluscs [65].

Moreover, a study has shown that insect protein extracts contain specific allergenic proteins that are predominantly present in insects but are much less abundant or absent in closely related organisms [65]. These specific insect allergens include chemosensory proteins, odorant- or pheromone-binding proteins, and hexamerin, the primary storage protein found in insect fat bodies. Additionally, three other proteins, apolipophorin III, larval cuticle protein, and the receptor for activated protein kinase, also serve as specific markers due to their predominant distribution in insects, with significantly lower occurrence in crustaceans and nematodes [65]. The cockroach allergen-like protein is distinct, as it only occurs in the yellow mealworm. This is important to ensure food safety, since these proteins can be used as probes to specifically detect insect proteins in various food products [65].

Epidemiological data and case reports of allergic reactions from insect consumption are still limited and often lack contextual information. The regions where insects are consumed are primarily rural, which might result in sub-notification, since many cases may only have been documented in the local literature [64]. However, some reports suggest that insects are responsible for 4.2–19.4% of food allergy cases in Asian countries, with silkworm pupae being a major cause of food allergies in China and Korea [64]. As edible insects are being introduced into the food markets of Western countries, more cases of food allergies are expected to be reported. Such cases are crucial in gaining a deeper understanding of primary sensitisation to edible insects, particularly concerning the key allergens involved and whether this sensitisation is specific to certain species [64].

### 7.6. Anti-Nutritional Factors

Anti-nutrients, also known as anti-nutritional factors, are food compounds that, when consumed in large quantities and over an extended period, reduce the human body’s bioavailability and utilisation of nutrients [66]. The content of anti-nutrients in insects varies widely, probably due to several factors, including the diverse chemical compositions of the plants that they consume [67]. Edible insects are mostly herbivores, feeding on plants and their parts that synthesise various secondary metabolites, which subsequently accumulate in the insects’ bodies [67].

The primary anti-nutrient compounds detected in edible insects include thiaminase, phytic acid, tannins, oxalates, saponins, quinones, phenolic compounds, cyanogenic glycosides, and toluene [2,66,68]. A study conducted a quantitative analysis of anti-nutrients in two edible insects from Zimbabwe—*Macrotermes facilger* and *Henicus whellani* [66]. These species primarily exhibited high concentrations of oxalates, which chelate calcium and magnesium, reducing or preventing their absorption [66,68]. The decreased availability of calcium negatively impacts bone formation, hormonal and enzymatic function, osmotic balance, and nerve impulse transmission [66,68]. Moreover, the prolonged ingestion of oxalates can lead to kidney stone formation (calcium oxalate crystals), as oxalates are filtered through the kidneys for excretion [66,68]. The above study revealed high oxalate levels in insects, ranging from 9.31 g/100 g for *Henicus whellani* to 14.08 g/100 g for *Macrotermes facilger* [66]. Phytic acid also reduces the absorption of essential minerals such as calcium, iron, magnesium, and zinc [66,68]. However, this study did not find phytate in any of the insect species analysed [66]. Regarding cyanogenic glycosides, these substances are known to interfere with cellular oxidation by binding to the catalytic ion of the enzyme cytochrome oxidase [2,66,68]. This interaction disrupts the transfer of electrons to oxygen molecules, ultimately inhibiting the cellular oxidation process [2,66,68]. In this study, cyanogenic glycoside tests were not performed in insects [66]. Nevertheless, low levels of cyanogenic glycosides (23–140 μg/100 g) have been reported in wild-harvested and processed *Eulepida mashona* and edible stinkbugs [2]. Finally, some compounds found in insects provide valuable functions in the human body but also exhibit anti-nutritional activity [68]. For example, tannins form complexes with iron, proteins, carbohydrates, and lipids, reducing the bioavailability of these nutrients [66,68]. Similarly, saponins can bind to zinc and iron [66,68].

Although the levels of anti-nutrients in commonly consumed insect species are generally very low, their effects can still be harmful, especially for people with poor diets and insufficient nutrient intake [2]. Therefore, it is crucial to adopt processing methods, such as boiling and cooking, to reduce the anti-nutrient levels in edible insects [66]. Furthermore, additional research is needed to determine the occurrence of these substances in a wider variety of insect species and to identify any other compounds that may be deleterious to human health [2].

## 8. Future Perspectives

Edible insects offer a promising and sustainable solution to meet the nutritional needs of the growing global population. Nonetheless, several challenges must be overcome to fully harness the potential of edible insects in improving the current food production system [2]. To optimise mass production technologies and reduce costs, enhancing innovation in mechanisation, automation, processing, and logistics is essential. This includes developing feeding tables, determining the nutritional value of substrates, conducting life cycle assessments, and maintaining genetic diversity to prevent colony collapse in insect farming [2]. For food and feed safety, it is crucial to investigate potential insect allergies in humans and chitin digestibility. Expanding data on the nutritional value of edible insects and their health benefits is essential. Additionally, researching the risks biological and chemical hazards, especially when using bio-waste streams, is vital. Developing methods to increase the shelf life is also important [69]. From a regulatory perspective, the establishment of voluntary guidelines and comprehensive legal frameworks governing the use of insects as food and feed is imperative. These should address human health and animal welfare considerations at both national and international scales. Additionally, advancing risk assessment approaches for large-scale farming and wild harvesting is critical to mitigate the risk of introducing non-native or invasive insect species into natural ecosystems [69]. Finally, ensuring consumer acceptance and education is crucial. Supporting entomophagy in cultures where it is prevalent, conducting comprehensive research on the ecology of consumable species, and educating consumers on the benefits of entomophagy are essential. Developing insect-based products, integrating insects into diets, and promoting insects as animal feed supplements are also vital steps [69].

An additional application of these animals is in pet food produced from insect protein. The use of insects in the pet food industry is supported by the evolutionary adaptation of pets’ wild ancestors to consume insects in their natural environments [70]. Additionally, the chemical composition of insects aligns with the nutritional needs of these animals [71]. In the EU, eight insect species are authorised for use in pet food. The top three most commonly farmed insect species for this purpose are *Hermetia illucens*, *Tenebrio molitor*, and *Acheta domesticus* [71]. However, it still remains a more expensive option compared to conventional pet food.

## 9. Conclusions

In a world where food excess and food scarcity coexist, it is imperative to act to eliminate these two opposing realities. This is the only way to ensure equitable access to essential goods without compromising the ability of future generations to meet their own needs. In this context, although entomophagy is an ancestral practice, it now emerges as a potential solution to some of the problems associated with the current food consumption patterns.

Edible insects have demonstrated a nutritional profile that is rich in macro- and micronutrients that are essential for the proper functioning of the human body. Their composition is often comparable to, and sometimes superior to, those of the most commonly consumed protein sources. However, nutrient bioavailability can vary widely; some studies report reduced digestibility or nutrient absorption due to chitin content, while others suggest that processing techniques can mitigate these effects. These scientific uncertainties underscore the need for further research to better understand the health impacts of edible insects. Regarding their health-related biological properties, edible insects have demonstrated various beneficial types of activity, such as antioxidant, anti-hypertensive, and anti-inflammatory effects, which can help to prevent the development of diseases. Nonetheless, most of this evidence comes from in vitro studies, and many of these effects have yet to be confirmed in clinical trials, representing a current limitation of this review. As expected, insect production is significantly more environmentally friendly than livestock farming, which is one of the main advantages of these foods. Furthermore, this practice is also associated with socioeconomic benefits for both developed and developing countries. It enhances food security for low-income families and creates business opportunities.

From a regulatory point of view, entomophagy has recently been regulated in the EU. Edible insects are classified as “novel foods” under the current Regulation (EU) 2015/2283, with commercialisation authorised only for a limited number of species. The high regulatory requirements for this type of food are designed to bring safe products to the market, but their complexity can be a barrier for investors, farmers, and entrepreneurs. The availability and consistency of regulatory data varies significantly across regions, with more comprehensive information available in high-income countries and limited or inaccessible data in others. This inherent disparity represents another limitation of the present study. Regarding food safety, edible insects generally have a safety profile that makes them suitable for human consumption, but further studies are needed to substantiate this type of claim.

Thus, by balancing the benefits of edible insects with an awareness of their limitations, entomophagy could be a promising approach to improving the current food system. To achieve this, we must change our predominantly negative attitude towards what is likely to be the “food of the future”.

## Data Availability

The original contributions presented in this study are included in the article. Further inquiries can be directed to the corresponding author.
